# Does the pronator-sparing approach improve functional outcome, compared to a standard volar approach, in volar plating of distal radius fractures? A prospective, randomized controlled trial

**DOI:** 10.1186/s10195-023-00700-y

**Published:** 2023-04-28

**Authors:** Gerhild Thalhammer, Laura A. Hruby, Theresia Dangl, Jonas Liebe, Jochen Erhart, Thomas Haider

**Affiliations:** 1 Department of Orthopedics and Trauma Surgery, Medical University of Vienna, Spitalgasse 23, 1090 Vienna, Austria; 2 Division of Orthopaedics and Traumatology, Cantonal Hospital Winterthur, 8401 Winterthur, Switzerland; 3 Department of Orthopedics and Traumatology, Hospital of the St. John of God Brothers Eisenstadt, Johannes Von Gott-Platz 1, 7000 Eisenstadt, Austria

**Keywords:** Distal radius fracture, Volar plating, Osteosynthesis, Distal radius, Pronator quadratus

## Abstract

**Background:**

This study aimed to compare functional outcomes of a volar plate osteosynthesis for distal radius fractures (DRF) performed with either a standard volar approach (SVA), which required detachment of the pronator quadratus muscle, or a pronator-sparing approach (PqSA).

**Materials and methods:**

This prospective randomized controlled study included 106 patients scheduled for volar plate osteosyntheses. Patients were allocated to either the SVA group (*n* = 53) or the PqSA group (*n* = 53). Patients were blinded to treatment until completion of the study. The primary outcome measure was the Patient-Rated Wrist Evaluation (PRWE). Secondary outcome parameters were the Disabilities of the Arm, Shoulder, and Hand (DASH) score and the Modified Mayo Wrist Score (MMWS). Follow-up examinations were performed at 8 weeks and 3, 6, and 12 months postoperatively.

**Results:**

Overall, 91 patients were included in the final analysis: 48 in the SVA group and 43 in the PqSA group. The two cohorts were not significantly different in demographic characteristics, including age, sex, injuries on the dominant side, type of injury, and fracture classification. We found significant differences between groups at 6 months in the mean PRWE (SVA: 12.3 ± 10.4, PqSA: 18.9 ± 14.11 points) and in the mean DASH score (SVA: 12.3 ± 11.9, PqSA: 19.3 ± 16.7 points), which favoured the SVA. We found no significant differences between groups in the MMWS or in the PRWE and DASH scores at any other time points.

**Conclusions:**

This randomized comparative clinical trial failed to demonstrate that a volar plate osteosynthesis performed with a PqSA could improve the outcome, compared to the SVA, in patients with DRF.

**Level of evidence:**

II

*Trial registration* Comparison of Two Volar Plating Systems for Distal Radius Fractures, ClinicalTrials.gov (NCT03474445), registered 22 March 2018, retrospectively registered, https://clinicaltrials.gov/ct2/show/NCT03474445?cond=radius&cntry=AT&draw=2&rank=1

## Introduction

Distal radius fracture (DRF) is the most common adult fracture. It is predicted that the projected increment in osteoporosis prevalence due to population ageing will increase the incidence of DRFs [1]. DRFs are commonly treated with surgical fixation; in most cases, a volar plate fixation is performed [2–4]. Most frequently, the Henry approach to the distal radius and modifications thereof are performed. In general, the skin is incised over the course of the flexor carpi radialis (FCR) tendon, and then access is developed between the FCR tendon and the radial artery. A possible modification of this approach is to open and prepare the FCR tendon sheath [5]. In both approaches, access to the volar aspect of the distal radius is typically achieved by detaching the superficial head of the pronator quadratus muscle (PQ).

The importance of the superficial head of the PQ is controversial. It was shown to be involved in forearm pronation [6]. In healthy individuals, inhibiting PQ function leads to reduced grip strength or pronation force [7]. Nonetheless, abundant studies have demonstrated that repairing the superficial head of the PQ did not improve functional outcome after a volar plate osteosynthesis for a DRF [8–12]. However, it is not always feasible to repair of the superficial head of the PQ due to injury-related or patient-specific factors. Additionally, functional repair might not be feasible due to its broad origin at the volar aspect of the distal radius.

Recently, PQ-sparing approaches have been postulated for volar plate osteosynthesis in DRF fractures [13–15]. However, the potential impact of sparing the PQ on the functional outcome of a volar plate osteosynthesis for a DRF remains poorly understood. The present prospective, randomized controlled study aimed to compare the standard volar approach (SVA), which requires the detachment and refixation of the PQ muscle, to a PQ-sparing approach (PqSA).

## Patients and methods

This study was conducted according to the principles of the Declaration of Helsinki. It was approved by the local institutional review board (protocol number 2339/2016). All patients provided written informed consent to participate. The study protocol was registered and uploaded at clinicaltrials.gov (NCT03474445).

In this study, we consecutively included patients with unstable distal radius fractures scheduled for volar plating at our department. Fractures with at least one of the following criteria were considered unstable: dorsal angulation > 20°; dorsal comminution; intra-articular radiocarpal fracture; associated ulna fracture [16]. The study included patients aged 18–75 years, with DRFs (AO types A2, A3, B1, B3, C1, C2, C3) scheduled for open reduction and internal fixation between March 2017 and August 2020. All but four fractures were closed. The four open fractures were I° according to the Gustilo–Anderson classification [17].

**Fig. 1:**
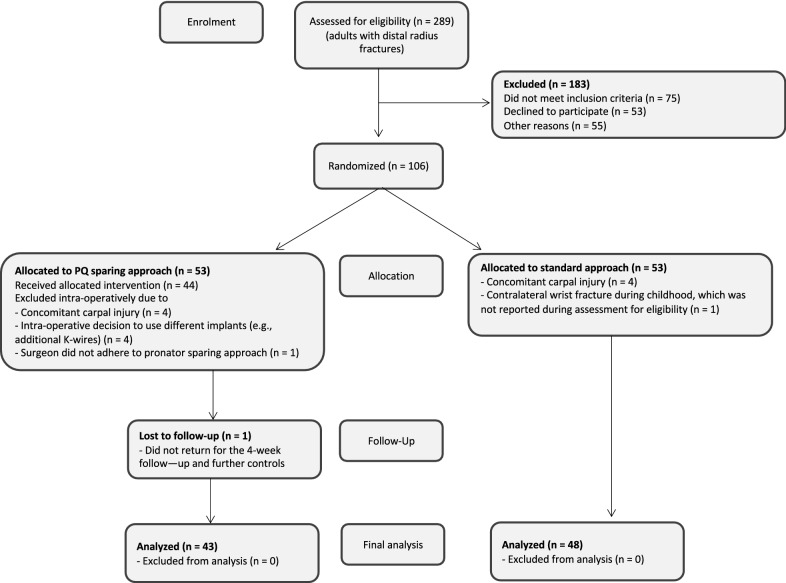
Consolidated Standards of Reporting Trials (CONSORT) flow chart showing the recruitment of patients with DRF. *PQ* pronator quadratus

Exclusion criteria were severe systemic disease (≥ ASA 3); polytraumas; previous trauma to the affected wrist and/or hand, including fractures during childhood and adolescence; associated carpal injury; previous injuries to the contralateral wrist, including fractures during childhood and adolescence; neurological disorders affecting the upper limb, including a history of carpal tunnel syndrome; cognitive deficits, including dementia; substance abuse; severe psychiatric disorders; non-adherence to the postoperative rehabilitation protocol; delayed definitive surgical treatment of more than 18 days after the injury; previous temporary surgical fixation (e.g. external fixation); signs and symptoms of complex regional pain syndrome (Fig. 1).

Upon written informed consent, patients were randomly allocated to SVA or PqSA treatment prior to surgery by picking a sealed envelope from a box locked in the principal investigator’s office. A person other than the operating surgeon picked each envelope. Patients were blinded to treatment until completion of the study. Irrespective of the allocated treatment modality, surgeries were performed by orthopaedic and trauma surgery residents undergoing training and under supervision by an attending surgeon; by attending surgeons in orthopaedic and trauma surgery; and by hand-specialized orthopaedic and trauma surgeons. Prior to study initiation, the principal investigator explained and demonstrated the PqSA to all participating surgeons.

**Fig. 2:**
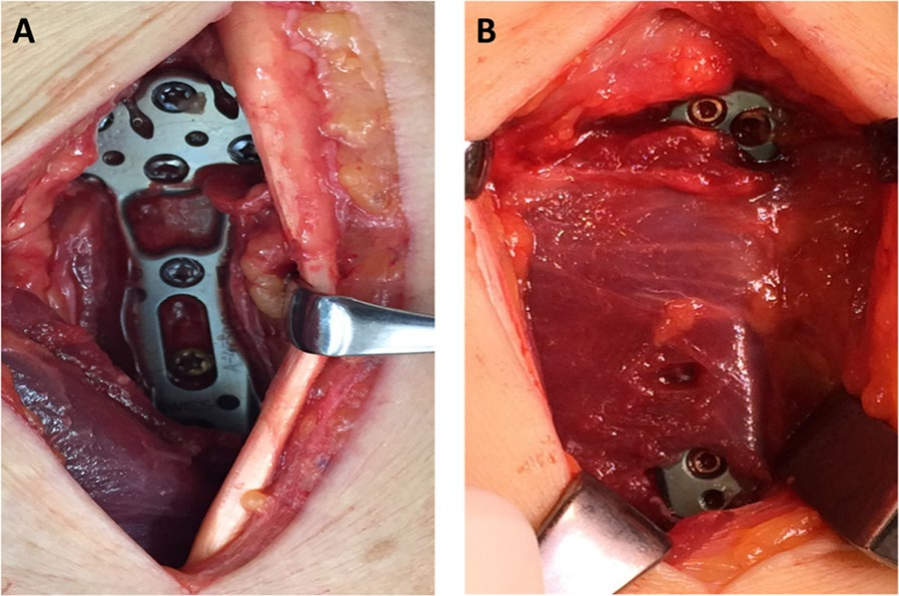
Intraoperative photographs of two approaches for treating distal radius fractures. **A** Standard volar approach; **B** pronator quadratus-sparing approach

The department’s SVA for a DRF was a trans-FCR approach. After visualizing the PQ muscle, the superficial head of the PQ was incised at the radial edge, and the muscle was lifted off the bone to visualize the fracture (Fig. 2a). After the fracture was reduced and the plate was mounted, the PQ muscle was refixed with 3–5 U-shaped stitches with a braided, absorbable synthetic suture (3.0 Polysorb, Medtronic Covidien, Austria). When the tissue quality (i.e. the remaining PQ muscle viability) was reduced due to preliminary damage and/or surgical handling, the PQ was not repaired. Patients in the SVA group received the department’s standard plating system (Distal Radius 2.5, Medartis®, Basel, Switzerland).

Identical to the SVA, the PqSA started with a trans-FCR approach. After carefully exposing the PQ muscle, a transverse incision was made just distal to the superficial head of the PQ, and the muscle was undermined to enable the sliding of the plate proximally underneath the muscle. The remaining steps were identical to those for the SVA. Screws were inserted through mini-incisions in the PQ, as required. To facilitate sliding the plate underneath the superficial head of the PQ, we used a plate with a low profile on the proximal plate end (INTEOS®—2.5 Radius Y-Plate, Hofer GmbH and Co KG, Austria; Fig. 2b).

All patients were immobilized with a forearm brace for 2–4 weeks, depending on the intra-operative bone stock, reduction quality, and surgeon preference. Depending on the timing of cast removal, physiotherapy was initiated at 2 or 4 weeks. This therapy focused on wrist/hand range of motion, strengthening, and activities of daily living, as per the institutional standard protocol.

Radiological and functional follow-up assessments were performed after 8 weeks, 3 months, 6 months, and 12 months. The primary outcome measure was the validated Patient-Rated Wrist Evaluation (PRWE) [18]. The PRWE was found to be the most sensitive outcome instrument for patients with DRFs [19]. This patient-related outcome measure rated wrist function in two (equally weighted) sections based on the patient’s experience of pain and disability. The score ranged from 0 to 100, where 100 was the worst pain [20]. Secondary outcome measures included the Disabilities of the Arm, Shoulder, and Hand (DASH) questionnaire and the examiner-based Modified Mayo Wrist score (MMWS) [21, 22].

All patients underwent standard radiographs and CT scans of the wrist pre-operatively. The standardized radiographic wrist assessment included posteroanterior and lateral projections. The CT scans (Somatom Edge plus, Siemens Healthineers, Germany) were performed in the prone position with the arm stretched over the head and the forearm and wrist in the neutral position. CT scans were evaluated with sagittal, coronal, and axial reconstructions in the bone window. Fractures were classified by three independent observers according to the AO/OTA classification [23], Fernandez classification [24], and Frykman classification [25]. Interobserver agreements were calculated. The results of this study were published elsewhere [26].

### Statistical analysis

The distributions of continuous variables were assessed with the Kolmogorov–Smirnov test. Mean values of variables with confirmed normal distributions in both treatment groups were compared with the two-sided *t*-test for independent samples. When at least one group lacked a normal distribution, the Mann–Whitney *U* test was performed. Numeric variables are expressed as the mean ± standard deviation (SD), unless stated otherwise. Sample size was calculated based on the desire to detect a 6-point difference in the PRWE score (SD ± 10) with a power of 0.8 and a tolerated *α* error of 0.05. The result indicated that 42 patients were required per group. We assumed a drop-out rate of 20% per group; thus, a total of 106 participants was required. In previous reports, the minimal clinically important difference (MCID) for the PRWE ranged from 6 to 14 points [27–30]. As a precautionary measure, we chose the lowest reported MCID for the PRWE score as the desired detectable difference in the sample size calculation. Secondary outcome measures, the DASH and MMWS, were evaluated without prior power analyses. Categorical variables were compared between the two groups with the chi-squared test. Per-protocol analysis was performed for all assessed variables.

Data were analysed with SPSS® statistics software (version 25, IBM®, USA). Data were visualized with Prism 9 (GraphPad Software, USA).

## Results

Ninety-one patients were included in the final analysis: 48 in the SVA group and 43 in the PqSA group. The two cohorts were not significantly different in age, sex, injuries on the dominant side, type of injury, number of type-I open fractures, and fracture classifications (Table 1). The mean age in both study groups was 50 years (range, 21–75). Female patients were predominant (SVA: 81%; PqSA: 86%). The majority of fractures were closed fractures (SVA: 94%; PqSA: 98%). The remaining fractures were type-I open fractures. The most common fracture type was C2 according to the AO classification (SVA: 35.4%; PqSA: 48.8%).

**Table 1:** Demographics of patients with distal radius fractures treated with either the standard volar approach or the pronator quadratus-sparing approach

Characteristic	Standard approach	PQ-sparing approach	*p* value
Number of patients, *n*	48	43	–
Mean age, years (range)	50 (21–75)	50 (21–75)	0.851
Women:men, *n* (%)	39 (81):9 (19)	37 (86):6 (14)	0.538
Fracture of dominant side, *n* (%)	28 (58):20 (42)	26 (60):17 (40)	0.836
Extension:flexion injury, *n* (%)	42 (88):6 (12)	37 (88):5 (12)	0.931
I° open:closed fracture, *n* (%)	3 (6):45 (94)	1 (2):42 (98)	0.362
AO classification			
A2, *n* (%)	2 (4.2)	0 (0)	0.498
A3, *n* (%)	7 (14.6)	4 (9.3)	
B1, *n* (%)	1 (2.1)	1 (2.3)	
B3, *n* (%)	1 (2.1)	3 (7.0)	
C1, *n* (%)	4 (8.3)	4 (9.3)	
C2, *n* (%)	17 (35.4)	21 (48.8)	
C3, *n* (%)	16 (33.3)	10 (23.3)	

*AO* Arbeitsgemeinschaft für Osteosynthesefragen, *PQ* pronator quadratus

### Primary outcome parameters

**Fig. 3:**
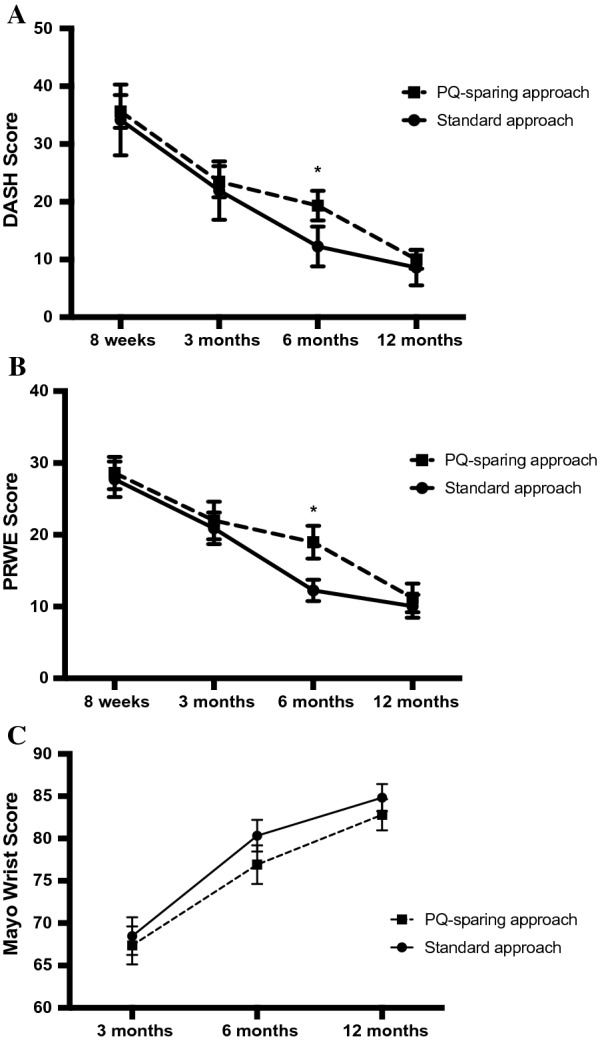
Graphs showing patient-reported evaluations of the outcomes of treatments for distal radius fractures. **A** The Patient-Rated Wrist Evaluation (PRWE); **B** the Disabilities of the Arm, Shoulder, and Hand (DASH) score; **C** the Modified Mayo Wrist Score. Scores were assessed at the indicated postoperative time points. *PQ* pronator quadratus

The PRWE measurements taken at different time points are shown in Fig. 3a. We found that the PRWE scores at 6 months significantly favoured the SVA (12.26 ± 10.39 vs 18.95 ± 15.11, *p* < 0.05), with a mean difference of 6.69 points between the two groups. However, PRWE scores were not significantly different between the cohorts at 8 weeks, 3 months, or 12 months (Table 2).

**Table 2:** Overview of clinical outcomes in patients with distal radius fractures treated with either the standard volar approach or the pronator quadratus-sparing approach

Clinical score			
Mean PRWE score (± SD)	Standard approach	PQ-sparing approach	*p* value
8 weeks	27.7 (17.2)	28.6 (14.7)	0.795
3 months	20.9 (15.3)	21.9 (17.1)	0.759
6 months	12.3 (10.4)	18.9 (15.11)	**< 0.05**
12 months	10.1 (11.0)	11.2 (13.1)	0.834

Mean DASH score (± SD)			
8 weeks	34.6 (21.1)	35.6 (18.7)	0.822
3 months	21.9 (17.5)	23.5 (17.7)	0.627
6 months	12.3 (11.9)	19.3 (16.7)	**< 0.05**
12 months	8.6 (10.6)	11.0 (10.7)	0.755

Mean Mayo Wrist Score (± SD)			
3 months	68.5 (15.5)	67.38 (14.7)	0.736
6 months	80.3 (13.0)	76.9 (14.9)	0.268
12 months	84.8 (11.2)	82.8 (12.1)	0.526

Bold values indicate *p*-value < 0.05

*FCR* flexor carpi radialis, *PQ* pronator quadratus, *SD* standard deviation, *PRWE* Patient-Rated Wrist Evaluation, *DASH* Disabilities of the Arm, Shoulder, and Hand score, *Mayo Wrist Score* Modified Mayo Wrist Score

### Secondary outcome parameters

The SVA group presented significantly lower DASH scores than the PqSA group at 6 months postoperatively (12.25 ± 11.86 vs 19.32 ± 16.73, *p* < 0.05). The mean difference between groups was 7.07 points, The groups were not significantly different in DASH scores at other time points (Table 2).

The groups showed no significant differences in MMWS scores at 3 months, 6 months, or 12 months (Fig. 3b, c).

There were no approach-related or hardware-related complications, including tendon ruptures, in either group. Furthermore, there were no infections or non-unions in either group.

## Discussion

In this randomized clinical trial for patients with DRFs, we compared clinical outcomes of treatment with volar locking plate fixation performed with either an SVA or a PqSA. We found that the SVA group showed significantly favourable PRWE and DASH scores, compared to the PqSA group, at 6 months after surgery. These scores were defined a priori as primary and secondary outcome parameters. At all other included time points, the PRWE, DASH, and MMWS values were not significantly different between the two study groups.

Since the volar approach has evolved into the standard surgical approach for treating DRFs, it has remained controversial as to whether the PQ muscle should be repaired [8, 10, 11, 31–34] and whether it should be spared from the start [13–15]. Proponents of the PQ repair and sparing approaches have argued that the muscle could protect the flexor tendons by covering the hardware, that pronation strength would be restored, and that distal radio-ulnar joint stability would be maintained and augmented [32–34]. On the other hand, opponents have claimed that tight closure of the muscle might lead to pain [11] and even to ischaemic contracture, which could result in limited forearm rotation [31].

Previous studies that compared PQ repair to non-repair did not find any differences in clinical outcome at 6 and 12 months postoperatively [8–11]. On the other hand, Tosti and Ilias demonstrated that patients displayed better grip strength and wrist flexion after PQ repair in early (at 6 weeks) postoperative clinical assessments [10]. A recent systematic review and meta-analysis by Shi and Ren included six studies that represented a total of 203 patients who received pronator repair and 180 patients who lacked pronator repair. They revealed that the two groups showed no significant differences in the DASH score or in pronation and grip strength [35].

A minimally invasive plate osteosynthesis combined with a pronator-sparing approach was first described by Sen and Harvey in 2008 [13]. Later, Dos Remedios et al. and Cannon et al. described the pronator-sparing approach combined with a standard FCR approach [14, 15]. The rationale behind introducing this approach was that, according to the authors, it required less soft tissue stripping, caused less flexor tendon stiffness postoperatively, and carried a lower risk of surgery-related complications compared to the standard approach. Those authors also suggested that potential benefits could include better pronation and grip strength, better stability of the distal radio-ulnar joint, and less scarring, which would result in a better range of motion. Based on these potential advantages, we hypothesized that a PqSA would yield better clinical outcomes than the SVA without PQ sparing.

Our results did not support our hypothesis. The two study groups showed no significant difference in the MMWS at any evaluated time point. The MMWS is a physician-based scoring system that evaluates pain, active wrist extension and flexion, and wrist grip strength (expressed as percentages of analogous measurements in the contralateral wrist) in addition to the ability to return to regular work and activities [22]. We found no differences in grip strength or range of motion between the two study groups. Therefore, we concluded that the PqSA did not influence the functional parameters of the wrist after a DRF. Fan et al. first compared the pronator-sparing and pronator-repair approaches in a comparable patient cohort [36]. They included patients with AO/OTA type A2 to C3 fractures who had a mean age of 42.5 years. They found that the pronator-sparing group had better grip strength, a greater range of motion in forearm rotation, and less wrist pain than the pronator-repair group at 1, 2, and 6 weeks postoperatively. However, consistent with our results, they found no significant differences in later assessments performed at 3 and 12 months. Another study by Itoh et al. that compared pronator sparing and pronator release and repair showed similar results [37]. In 65 patients with AO/OTA type C2 and C3 fractures, they assessed range of motion for wrist flexion and extension, forearm rotation, percentage of grip strength compared to the contralateral uninjured wrist, and pain at six different time points. Their results showed no significant differences in any functional parameters, except that the pronator-sparing group reported significantly lower pain scores at 2, 3, and 4 months postoperatively.

In this study, the PRWE was the primary patient-reported outcome measure. Originally described by MacDermid et al. in 1998 [18], the PRWE is a reliable tool for quantifying pain and disability after DRFs, and it was found to be the most sensitive outcome instrument for patients treated for DRFs [19]. We found that the PRWE revealed a significantly better score in the PqSA group at 6 months, but no significant difference was observed at any other time point. At 6 months, the absolute mean difference in the PRWE was 6.6 points, which was at the lower end of the previously published MCID (6–14 points) for the PRWE [27, 28, 30, 38]. While statistically different, the difference is most likely not clinically meaningful. At 6 months, we also found a significant difference (mean: 7 points) in the DASH scores between the two study groups which favoured the PqSA group. This value was below the previously published MCID (10–13.5 points) for the DASH score [30, 38]. Previous comparative studies showed that the sparing approach resulted in better clinical outcomes. For example, Fan et al. showed significantly better DASH values in the pronator-sparing group after 6 weeks, but only minimal, insignificant differences after 3 and 12 months [36]. They reported DASH scores of 26.3 in the repair group and 18.4 in the pronator-sparing group, which were below the values we measured at 8 weeks. However, similar to our results, the 8-point difference between their two groups was below the published MCID for the DASH score [30, 38]. That study did not include an evaluation at 6 months postoperatively. Itoh et al. also found significantly lower QuickDASH scores in the pronator-sparing group after 1 and 2 months [37]. Moreover, the difference was within the range of the published MCID (8–19 points) for the QuickDASH score [28, 30]. Nevertheless, they also failed to detect significant differences in the assessed clinical outcome parameters at a later postoperative time point.

This study had some limitations. As opposed to previous studies [36, 37], we did not assess clinical outcome parameters or patient-rated outcome measures in the early postoperative period. Therefore, we could not determine whether the PqSA was associated with favourable results at early time points, as shown in previous studies. Previous studies showed that the superficial head of the pronator quadratus muscle is the prime mover of forearm pronation [39]. Therefore, it would have been useful to measure the pronation torque in addition to the collected parameters. That measurement might have demonstrated a positive effect of the PqSA. Indeed, Armangil et al. previously demonstrated that inhibiting PQ function led to an 18.5% reduction in pronation strength [40]. A similar result was found by McConkey et al. [7], who used lidocaine to paralyse the PQ of healthy subjects and found that pronation torque was reduced by 21%. In contrast, studies by Ahsan and Yao [41] and Huh et al. [42] reported that a pronator muscle detachment had no effect on grip strength or pronation. Another study limitation was that our study included multiple surgeons and two different plate systems, which might have influenced the study results.

In conclusion, this randomized comparative clinical trial failed to demonstrate clinically relevant differences in outcomes between the PqSA and SVA for volar plate osteosynthesis in patients with DRFs.

## Data Availability

The dataset analysed in this study is available from the corresponding author on request.

## Abbreviations

DRF: Distal radius fractures
SVA: Standard volar approach
PqSA: Pronator-sparing approach
PRWE: Patient-Rated Wrist Evaluation
DASH: Disabilities of the Arm, Shoulder, and Hand
MMWS: Modified Mayo Wrist Score
FCR: Flexor carpi radialis
PQ: Pronator quadratus muscle
SD: Standard deviation
MCID: Minimal clinically important difference
